# Solid Particle Erosion Studies of Varying Tow-Scale Carbon Fibre-Reinforced Polymer Composites

**DOI:** 10.3390/ma15217534

**Published:** 2022-10-27

**Authors:** Suresh Kumar Shanmugam, Thirumalai Kumaran Sundaresan, Temel Varol, Rendi Kurniawan

**Affiliations:** 1 Faculty of Mechanical Engineering, Kalasalingam Academy of Research and Education, Krishnankoil 626126, Tamil Nadu, India; 2 Department of Mechanical Engineering, PSG Institute of Technology and Applied Research, Coimbatore 641062, Tamil Nadu, India; 3 Department of Metallurgical and Materials Engineering, Karadeniz Technical University, Trabzon 61080, Turkey; 4 School of Mechanical Engineering, Yeungnam University, 280 Daehak-ro, Gyeongsan-si 38541, Gyeongsangbuk-do, Korea

**Keywords:** erosion, roughness, tow size, carbon fibre-reinforced polymer composite (CFRP)

## Abstract

Solid particle erosion inevitably occurs if a gas–solid or liquid–solid mixture is in contact with a surface, e.g., in pneumatic conveyors. Nowadays, an erosive failure of the component after the usage of a long period has been gaining the interest of the researchers. In this research work, carbon fibre-reinforced polymer (CFRP) composites are prepared by varying the tow sizes of fibres, such as 5k, 10k, and 15k. The prepared composites are subjected to erosion studies by varying the process parameters, such as the impact angle (30, 60, and 90 degrees) and velocity (72, 100, and 129 m/s). The Taguchi orthogonal array design has been employed for the experimental plan and the erosion rate and surface roughness are observed for each run. The changes in the responses are reported for varying process parameters. The higher erodent velocity of 129m/s leads to higher erosion rates and forms poor surface quality. The minimum impact angle of 30 degrees provides higher erosion rates and higher surface roughness than the other impingement angles. Finally, the eroded surface of each sample is examined through microscopic and 3D profilometer images and the erosion mechanism is analysed at different conditions. The eroded particles supplied at lower speeds do not penetrate the composite surface. However, it is well-known that the lower the collision force, the harder the traces on the surface, yet no sign of fibre breaking or pull-out is observed. The passage of erodent particles on the composite caused surface waviness (flow trace), which prevents the surface from degrading.

## 1. Introduction

Materials are the key factors in engineering, since the desired materials are required to meet the needs of the environment that they are meant for use in. Engineering materials are generally grouped into two parts, i.e., metals and non-metals. However, there are many other engineering materials, such as ceramics, polymers, semiconductors, composites, biomaterials, and advanced materials [1]. Polymers are large molecules made up of repeating small, simple chemical units. In some cases, the iterations are linear, such as a chain made up of its links. In other cases, the chains are branched or linked to form a 3D network. The repeating units of the polymer are usually the same or nearly the same as the monomers or the origin materials from which the polymer is formed. Further, the materials possess their own merits and demerits. However, the need for lightweight materials has been continuously increasing for various applications, such as in automobile and aerospace industries [2].

Replacing the metals with polymer composites is the prime aim of most researchers since the polymer composites are lightweight materials. Various carbon fibre-reinforced polymer (CFRP) composites can be substituted for metals, with excellent specific stiffness, strength, and good corrosion resistance [3,4]. As a kind of CFRP, short-carbon fibre-reinforced polymer (SCFRP) has more stable properties under complex loading conditions because of the randomly oriented short fibres, and hence, the gap between the continuous-fibre composites and the unreinforced polymers [5] is filled. Thus, SCFRP has been widely used in automotive, aircraft, and aerospace industries [6]. To further improve the performance of these composite materials, several existing studies have discussed the influence of oxidised fibres, matrix properties, fibre aspect ratio, and volume fraction on the mechanical properties of composites [7,8]. However, in many such engineering applications, the mechanical behaviour and erosion characteristics of the polymer composites are the main requirements. To cater to such needs, the polymer material should have better properties.

Solid particle erosion (SPE) is a repetitive process that removes the material from a surface, due to solid particle impacts. It happens wherever solid particles, e.g., sands, metallic particles, and foods, are carried out in a conveying system by liquids or gases. Several industries, such as energy producers, pharmaceuticals, and chemical engineering, have employed conveying systems. SPE may damage the conveyors, and hence, they require regular maintenance. Sometimes, substantial harm is caused to the system due to total failure of parts. Therefore, by estimating the erosion in the conveying systems, one can optimise the operating conditions to attain better designs and parts, for a longer lifetime, and maintenance and production costs can be reduced [9]. The polymer composites are extensively affected by erosion damage due to their extended use in hostile environmental applications [10]. However, there is an intricacy in that the polymers have poor erosion wear behaviour owing to their brittleness. Since the surfaces of diverse products and materials are constantly subjected to destructively erosive circumstances, erosion is a critical concern in many modern industries [11]. 

One of the many types of degradation classified as wear is the erosion of materials caused by the surface impact of hard particles. It is a complicated phenomenon that frequently involves mechanical, chemical, and material parameters. This complexity appears to defy simplification on the part of both the experimentalist seeking to carefully separate variables and the theorist attempting to accurately model the wear system. However, significant progress has been made in recent years in both gaining a basic understanding of the significant parameters of wear and applying a materials methodology to mitigate wear problems [12]. On the other hand, the erosion wear is mostly influenced by the erosion process parameters, such as erodent velocity, impingement angle, and erosion particle size. Most of the researchers have performed experimental work on metals and ceramics. The reported research works are single-particle erosion, multiple-particle erosion, various theories of erosion, and their environmental effects. However, in recent decades, the interest has changed towards new materials, such as composite materials. Polymer-based composites were one of the focuses of the researchers as they are used in various applications. Moreover, the various kinds of reinforcements used for the composite fabrication also play a role on affecting the erosion behaviour of the materials [13,14]. Jena and Satapathy [15] have investigated the erosion property of fabricated polymer composites which are filled with red mud and glass. The experimental result has revealed that the weight proportion of filler, weight percentage of fibre, erodent velocity, and impact angle are important parameters which significantly contribute to minimising the wear rate of the composites. The erosion rates of the jute and glass fibre-reinforced epoxy-based composites have been analysed by Patel et al. [16]. The process parameters considered for the experimental analysis are impingement angle, particle speed, and erodent size, with an objective to measure the erosion rate. At the higher impact angle of 90°, the brittle property of the composite is revealed. The notable changes in the erosion rate are also observed when the fibre weight and layering sequence are changed.

It is observed that the maximum erosion is revealed while testing the polymer composites at higher impingement angles. When the erodent flows in the same direction as the fibre orientation in the matrix, the erosion rate becomes less, and vice versa. Moreover, a low erosion rate is noted for the effective adhesion between the fibre and the matrix [17]. The bonding of the matrix and the fibre influences the erosion rate more, irrespective of the material [18]. Debnath et al. have conducted an experimental analysis on erosion rates of glass, carbon, and jute fibre-reinforced polyester composites. The result shows that the increased impact angle and erodent velocity increase the erosion rate. The decrement of erosion wear is noticed with an increased standoff distance, as it reduces the kinetic energy of the particle striking the material [19]. Patnaik et al. have attempted to optimise the erosion rate of a glass fibre- and alumina-reinforced hybrid composite. Upon increasing the velocity and particle size, the erosion rate is observed to be maximised. The analysis shows that the particle size has a higher influence on affecting the erosion wear than other parameters. The repeated impact of erodent particles on the material surface reveals surface cracks [20].

Suihkonen et al. have produced glass fibre-reinforced vinyl ester-based composites and attempted to determine their erosive wear behaviour using the slurry erosion wear test rig. The experimental results have exhibited that the tested rubbers have better wear resistance than the fabricated composite materials [21]. Zhang et al. have examined the wear resistance of carbon short-fibre-reinforced epoxy matrix-based polymer composites. The result reveals that the short-fibre reinforced in the matrix shows superior resistance compared to the others, owing to improved bonding [22]. Rao et al. have compared the erosion rate of a short poultry feather-reinforced epoxy-based polymer composite with pure epoxy polymers. The analysis has shown that poor mechanical properties are observed with the feather-reinforced composites, but the erosion resistance is improved to a significant level [23]. Tarodiya and Levy have developed a model for predicting the erosion of polymer-based composites and to minimise the time elapsed for the experimental study [24]. Arani et al. have used the angular silicon carbide particles as an erodent to compare the erosive nature of rubber particle-reinforced epoxy composites with neat epoxy. The results have revealed that the erosion rate of rubber particle-reinforced epoxy composites is lower than that of the neat epoxy [25].

From the literature review, it is clear from the existing experimental research that the importance of conducting an erosion study on various fibre-reinforced polymer composite is inevitable, as it has been suggested for various engineering applications. Most of the researchers have interest in finding the significant relationship existing between various erosion process variables and erosion resistance of metals and alloys only. However, limited research works have focused on finding the erosion performance of carbon fibre-reinforced polyester matrix composites. Still, the suitability of utilising carbon fibre-reinforced polymer composites for engineering applications needs to be ensured. The changes in the erosion performance of polymer composites for varying process inputs are also required to meet the needs of the industries. Hence, the need exists to focus on the performance of such CFRPs. Moreover, the surface quality of the component is also of interest to the researchers as it is important while fabricating the component used in such erosive environments. The primary aim of the present work is to provide a systematic experimental evaluation of the influence of the tow size (k) of the carbon fibre matrix on mechanical and erosion behaviours of the composite. In addition, this work examines the mechanism of erosion prevailing on the surface of the composite at varying conditions through an optical profilometer.

## 2. Experimental Work

### 2.1. Materials and Composite Preparation

The materials employed in this investigation were PAN-based carbon fibre as reinforcement, supplied by Dowaksa Company, Turkey, and the polyester resin as a matrix for hand-layup composite material fabrication.

**Figure 1:**
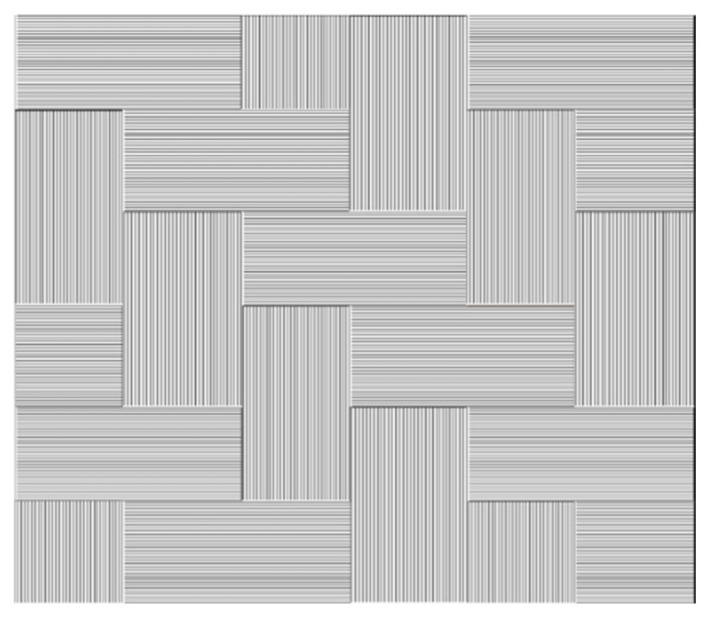
Representation diagram of 2 × 2 twill weave pattern.

The fibres were never twisted, and the fabric was a 600 gsm twill-woven fibre fabric with a 45 percent fibre weight fraction, and four layers of lamina were piled at a 0.135 mm thickness. Table 1 shows the fabric characteristics and typical carbon fibre properties. The resin and the hardener mixture were placed onto the fibres, and the die was closed and compressed to 17 MPa for 24 h for proper curing at room temperature. Then, 2 × 2 twill-woven composites with varied tow sizes (5k, 10k, and 15k) were produced, and the twill-woven representation diagram is demonstrated in Figure 1.

The mechanical properties were measured using an Instron (Series-3382) according to ASTM standards D3039-08 and D790-10, using a 100 mm gauge length and a 200 × 20 × 3 mm specimen sample. The impact strength of an unnotched specimen of 63 × 13 × 3 mm was tested using a 3.56 kg impact hammer on a Charpy impact tester in accordance with ASTM standard D256-10. Hardness has been evaluated in three distinct locations using a Rockwell hardness testing machine with a maximum load of 60 kg and a specimen dimension of 15 × 15 mm. In each test, the averages of three samples were analysed and reported. Mechanical properties of the composite laminate were tested in accordance with ASTM standards using the samples of the specified dimensions. Tensile and flexural strengths were also tested.

### 2.2. Erosion Experiment

The ASTM G76-13 solid particle erosion test has been carried out by utilising an air jet erosion tester (TR 470 of DUCOM, Bengaluru, Karnataka, India). The air jet erosion tester is made up of the following components: an air compressor, a feeder unit, a mixing chamber, a control unit, an erosion unit, and a dust collector. The erodent was alumina, irregular in shape, with an average size of 50 micron, and it was stored in the hopper as well as transported to the mixing chamber by the conveyor belt unit. The erodent was mixed with dry, compressed, high-speed air of 0.2–5 bar in the mixing chamber. After mixing, the air–erodent combination was pushed via the erosion unit’s tungsten carbide nozzle (1.5 mm diameter, 50 mm length) and the specimen was struck at high velocity. The sample was made to be 25 × 25 × 5 mm in size for the air jet erosion experiment. The experiment has been carried out with a continuous erosion time of 10 min and a standoff distance of 10mm. Each specimen was cleaned and weighted using a precision balance with 0.1 mg accuracy before and after the erosion test. Each experiment was performed three times, and the average weight loss was measured, then the erosion rate of each sample was computed using the weight loss. The erosion rate is the amount of material lost from the composite per unit time by the impact of the erodent released, and it may be computed using Equation (1) [26]:

Erosion rate = Weight loss discharge × time (g/g)(1)

The sample holder of varying impingement angles was employed to change the impact angle for the experimental work. By adjusting the air pressure, the erodent velocity was determined using the double-disc method. In terms of mechanical properties and for comparison, samples of carbon fibre-reinforced composites with varied tow sizes (5k, 10k, and 15k) were employed for erosion testing.

### 2.3. Experimental Plan

**Figure 2:**
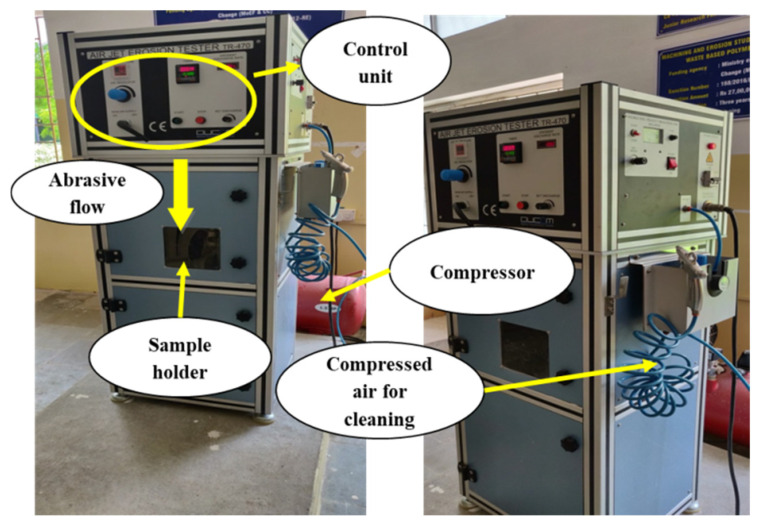
Erosion test facility.

When a significant number of elements are involved, statistical tools have been proven to play a vital role in assessing the product or process characteristics. Erosion is a frequent phenomenon in which the combined reaction of many control components influences the responses. The applications of various statistical approaches to investigate the influence of parameters are demonstrated in the existing literature. For the current experimental task, the Taguchi experimental design has been utilised to plan the experimental runs and to examine the importance of control variables in the output response. For the experiment, a typical L9 orthogonal array was used. Thus, by utilising Taguchi’s factorial experimental design [27], the number of experiments was minimised. The prepared sample was subjected to a solid abrasive particle erosion test (Figure 2), and Table 2 shows the process variables involved as well as their specified ranges.

The mean response of the experimental data was computed by translating the output data (erosion rate) to a signal-to-noise ratio (S/N). As the erosion rate should be as low as feasible, the erosion rate’s S/N ratio was judged to be “the smaller the better”. The ratio value for the “the smaller the better” criterion is stated as Equation (2) [28], which is a logarithmic modification of the loss function:

(2) $$\frac{s}{n}=-10\;\log\left[\frac{1}{n}\right]\sum y^{2}$$

In the preceding equation, *n* represents the number of observations, and *y* represents the observed data. The equation has been used to calculate the optimal state of the erosion rate and surface roughness, with the erosion control parameters set to the minimum. Finally, an ANOVA test was run to examine the significant effect of specific parameters on the erosion rate.

## 3. Results and Discussion

### 3.1. Physical and Mechanical Properties

The fabricated composites were subjected to testing and the determined properties are shown in Table 3. The tensile strength and the flexural strength of the composites were found to increase when the tow size of carbon fibre was increased to 10k, and then they decreased. Similarly, the hardness of the composites also started to decrease for the tow sizes of 10k to 15k. The maximum strength was noticed for carbon fibre of a 10k tow size, which ensured better bonding between the carbon fibres and the matrix materials. However, the presence of more fibres in the matrix led to poor bonding between the matrix and the fibres. In addition, a gradual increment of the impact strength of the composite was noticed for the change in tow size from 5k to 10k. There wasno change in the impact strength when the tow size was increased beyond 10k.

### 3.2. Effect of Parameters on Erosion Rate

The samples were tested using the solid particle erosion test rig by varying the parameters based on the standard orthogonal array system. The weight loss of the sample for each experimental run was measured and the erosion rate was calculated by using Equation (1). The surface roughness tester has been used to measure the surface roughness, and average roughness values are presented in Table 4. The experimental results were analysed using the Minitab 19 software tool.

**Figure 3:**
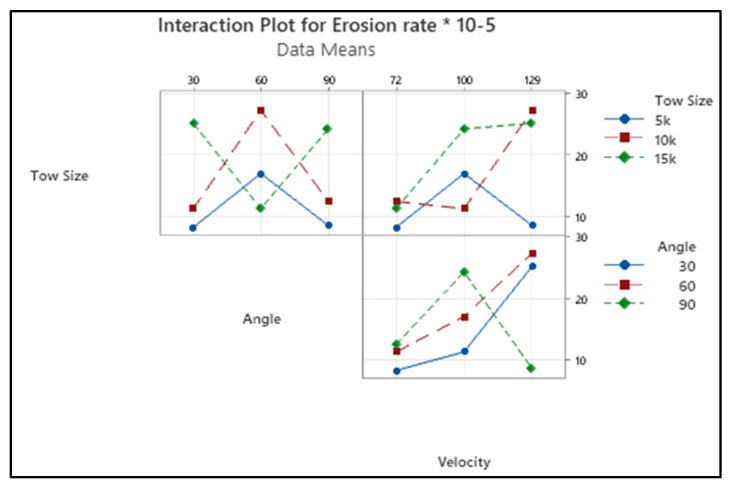
Interaction plot for erosion rate.

The erosion testing parameters and the material qualities had an impact on the solid particle erosion test. The solid particle erosion test on polymer composites heavily influenced the erosion test parameters, such as the impingement angle, erodent velocity, and erodent flow rate. The proper selection of these variables decides the performance, such as lower erosion rate and surface roughness. Figure 3 presents the interaction plot of the erosion rate of the chosen input parameters. The erosion rate decreased with the increased impact angle. It is generally known that for ductile materials, erosion occurs at lower impact angles, whereas for brittle materials, erosion occurs above 90° impact angles [14]. In this research work, the maximum erosion occurred on the fibres at 60°, velocity of 129 m/s, and a 10k tow size. The reason for the changes in the erosion rate of the composite material is the conversion of material to that of a semi-ductile nature. Similar results were observed by the researchers [29,30].

The composite erosion rate increased as the erodent velocity increased. When the hard abrasive particles strike the material surface at high speed, their kinetic energy is transferred into the impinging area and deforms the material plastically as well as separates the reinforcement from the matrix. However, the surface damage occurs on the composite surface as a result of the creation of micro-cuts, and craters are caused by the repetitive attack of hard erodent particles at a greater erodent velocity. Similarly, increasing the tow size increased the erosion rate of the composite.

**Figure 4:**
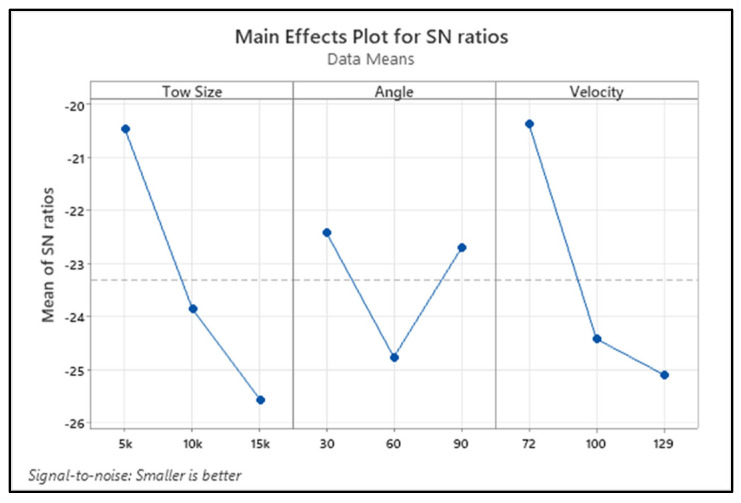
Main effect plot of the S/N ratio for erosion rate.

Figure 4 shows the variation of the erosion rate for the changes in the process variables in terms of the S/N ratio of the response characteristic. The delta term in Table 5 illustrates the significance of each process parameter. The higher the delta value, the more significance those parameters have in affecting the response values. It is understood from Table 5 that the tow size of the carbon fibre is the significant factor influencing the erosion rate. The erosion rate is the second parameter, followed by impact angle, and it significantly influences the erosion rate of the composite.

An analysis of variance (ANOVA) test has been carried out to understand the significance of each process parameter and their contribution to the output responses. Table 6 depicts the ANOVA results for the experimental results of the erosion rate. It is clear from the table that the tow size of the fibre contributes the most (45.23%) to influencing the erosion rate, followed by erodent velocity (43.78%) and impact angle (10.98%). Moreover, F-values of all the parameters were also higher, as per the F-test, and they indicate the consequence of corresponding factors affecting the particular responses.

### 3.3. Effect of Parameters on Surface Roughness

**Figure 5:**
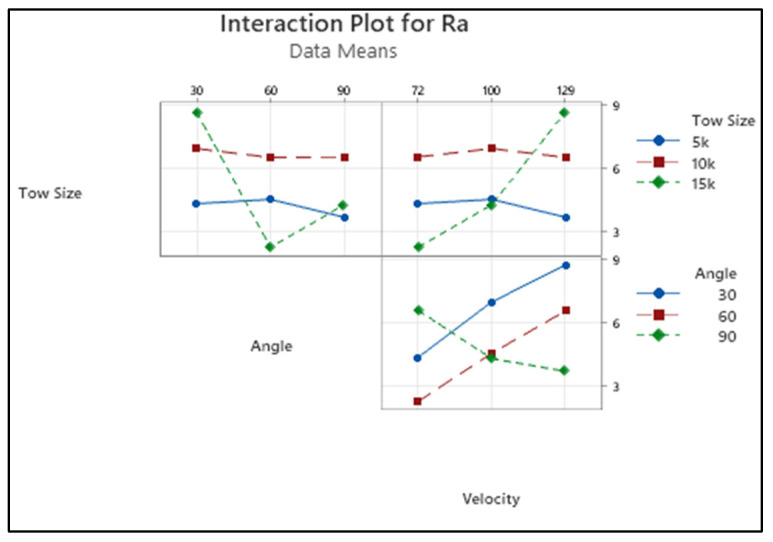
Interaction plot for R_a_.

Figure 5 shows the interaction plot of surface roughness for varying process parameters. The R_a_ value measured on the composite material tended to increase when the tow size increased from 5k to 10k. Again, a decrement was noticed for the change in tow size from 10k to 15k. This was caused by the effective package of fibre in the matrix, and it led to protecting the impact impinged by the solid particle. A decrement was observed while increasing the impact angle from 30 to 90 degrees.

The impact of hard particles on the surface in the perpendicular direction broke the fibre and formed the waviness [31]. When the composite surface was placed at a certain angle and the impact was at an inclined degree, the area on which the impact was caused was wider and deeper, and it resulted in the formation of craters on the composite materials. Sometimes, it also led to the formation of craters in the poor-quality region. Ultimately, the impact at certain angles damaged the surfaces and broke the fibres, as well as formed wider and deeper impacts with increased roughness.

The changes in the surface roughness were observed for varying erodent velocities, and they showed an increasing trend. Further, continuously increasing roughness was observed while increasing the velocity from 72 to 129 m/s. The fast-moving solid particle with kinetic energy was utilised for the removal of material from the composite. Here, the increased erodent velocity facilitated higher energy, which caused impacts on the surface of the material in larger quantities. On the other hand, it led to a higher number of small craters on the surface of the element. The continuous impact caused by the fast-moving solid particle led to the formation and clubbing of small craters, and wider, deeper craters on the surfaces. Subsequently, the surface roughness increased. Higher surface roughness was observed at an impact angle of 30 degrees and erodent velocity of 129 m/s.

The eroded surface texture was measured using a profilometer. R_a_ was observed for all the experimental runs. As the roughness on the eroded component is required to be minimum, “the smaller the better”, the objective function was also used for calculating the S/N ratio for the surface roughness.

**Figure 6:**
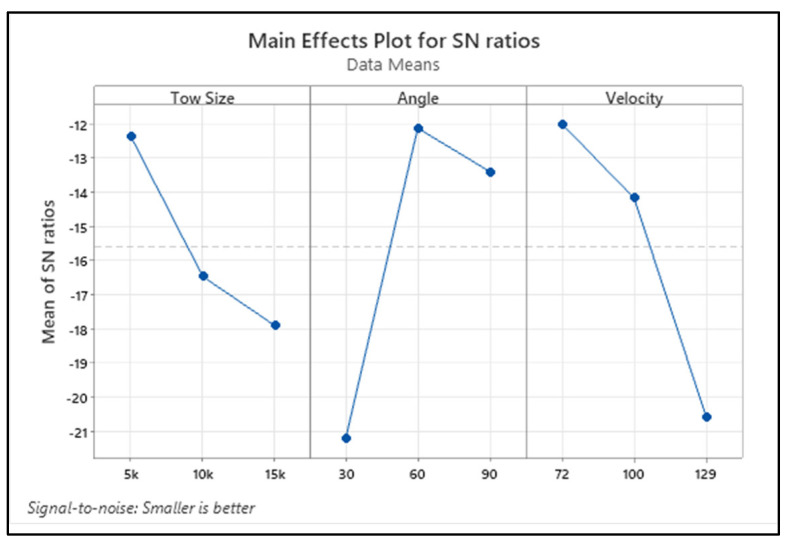
Main effect plot of S/N ratio for R_a_.

Figure 6 depicts the changes in roughness measured on the eroded samples with input process variables in terms of the S/N ratio and the mean of the response characteristic. It is understood from Table 7 that the delta value of the factor angle was higher than for the other two parameters. This implies the significant influence of the angle on the variation of roughness, followed by velocity and tow size.

From Table 8, it is clearly visible that all the parameters significantly contributed to affecting the surface roughness quantity. The impact angle contributed the most, i.e., the influence of the angle was higher (46.3%), to affecting R_a_, followed by erodent velocity and tow size factors. Moreover, F-values of all the parameters were also higher as per the F-test. It indicates the consequence of corresponding factors affecting the particular responses, and they were highly correlated.

### 3.4. Surface Topography Analysis

**Figure 7:**
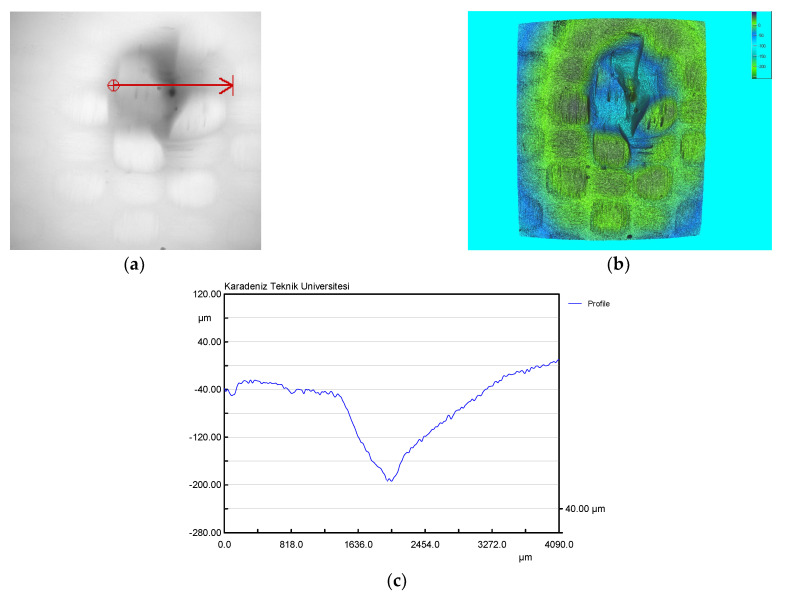
Surface of the eroded sample (Tow = 10k, Angle = 90 degrees, Velocity = 129 m/s), surface topography: (**a**) microscopic view, (**b**) 3D surface profilometer image, and (**c**) surface profile.

The surface topography has been examined using an optical microscope (Olympus CX-21i, with a continuous 10× magnification) and a 3D surface profilometer (Nano System NV-2000). The eroded surface of the composite was examined under varying conditions after the experimental work. The microscopic view depicts the scanning direction of the surface profile. The surface profile shows the eroded zone thoroughly with width and depth. The microscopic view, 3D profilometer image, and the surface profile observed on the eroded surface for the condition of a tow size value of 10k, angle at 90 degrees, and velocity of 129 m/s are shown in Figure 7. The surface roughness values measured were as follows: R_a_ = 27.032 µm, R_z_ = 46.588 µm, R_t_ = 143.930 µm, and R_q_ = 35.721 µm. The variations in the surface profile and 3D image exhibit the surface topography of the sample after the erosion test. The microscopic image reveals the deformation region caused by the erodent particle’s sliding movement.

The surface topography has been examined using an optical microscope (Olympus CX-21i, with a continuous 10× magnification) and a 3D surface profilometer (Nano System NV-2000). The eroded surface of the composite was examined under varying conditions after the experimental work. The microscopic view depicts the scanning direction of the surface profile. The surface profile shows the eroded zone thoroughly with width and depth. The microscopic view, 3D profilometer image, and the surface profile observed on the eroded surface for the condition of a tow size value of 10k, angle at 90 degrees, and velocity of 129 m/s are shown in Figure 7. The surface roughness values measured were as follows: R_a_ = 27.032 µm, R_z_ = 46.588 µm, R_t_= 143.930 µm, and R_q_ = 35.721 µm. The variations in the surface profile and 3D image exhibit the surface topography of the sample after the erosion test. The microscopic image reveals the deformation region caused by the erodent particle’s sliding movement.

The parametric influences affecting the response characteristics are understood from the experimental results and the interaction plot. Besides, the surface-level changes in the composite material caused by the erosive action of the sliding particle also need to be addressed. The physical surface of the component is also important in various applications, as it firmly develops contacts with its counterpart. Since the erosive action affects the surface texture of the component during the erosion test, the examination of surface topography of the sample is also equally important for understanding the parametric influence of process parameters.

**Figure 8:**
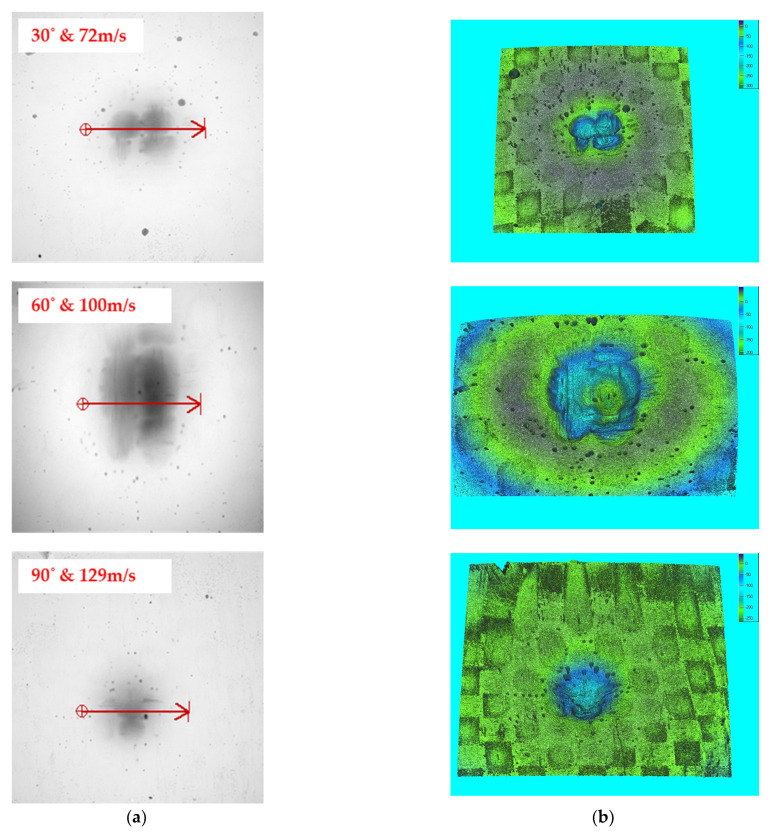
Eroded surface view for 5k samples: (**a**) microscopic view and (**b**) 3D surface profilometer image.

**Figure 9:**
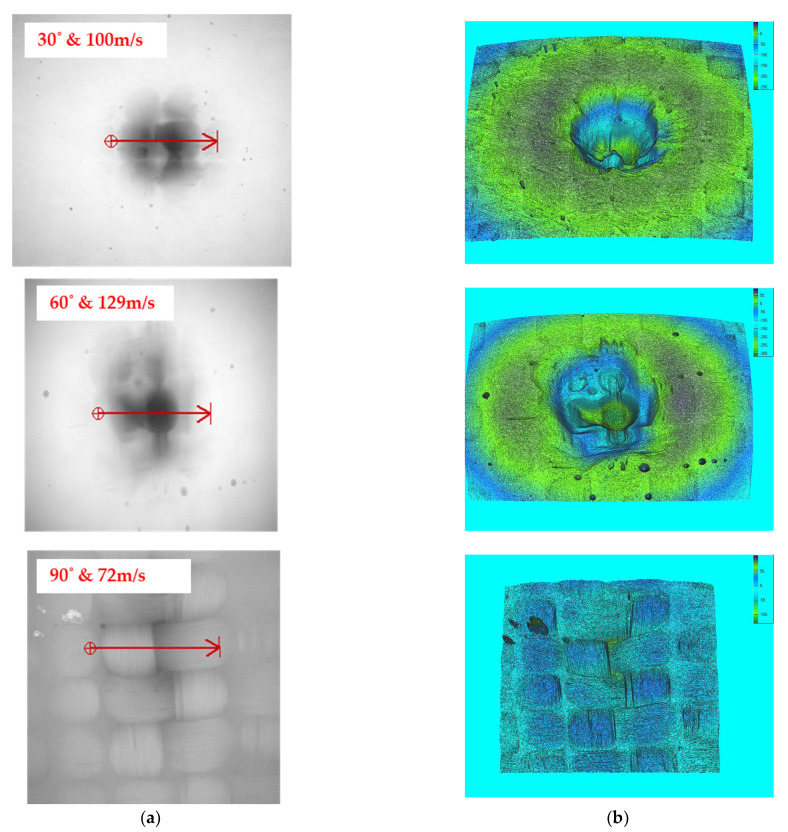
Eroded surface view for 10k samples: (**a**) microscopic view and (**b**) 3D surface profilometer image.

**Figure 10:**
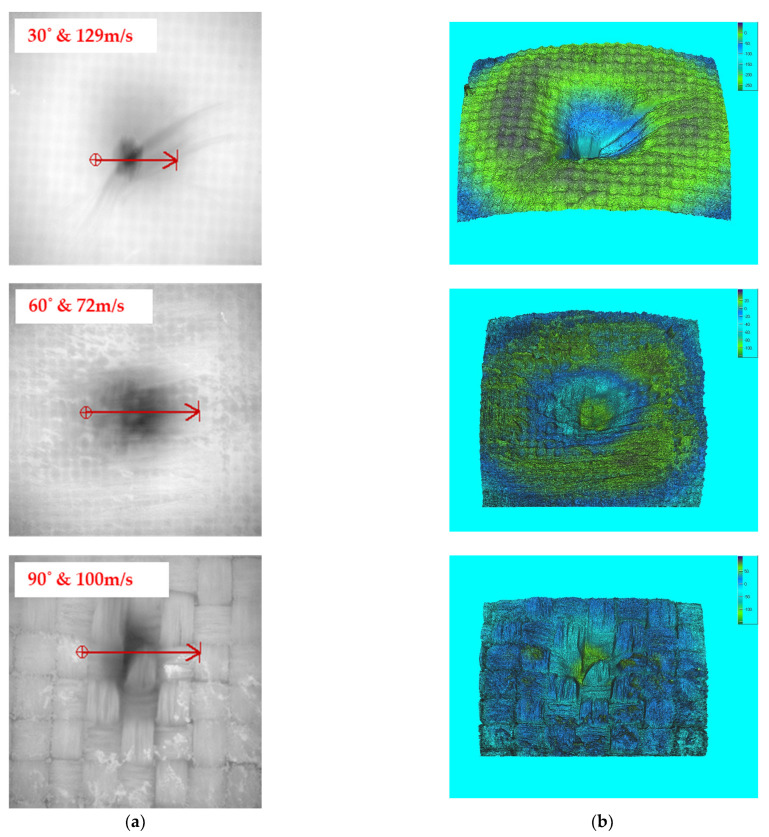
Eroded surface view for 15k samples: (**a**) microscopic view and (**b**) 3D surface profilometer image.

Bonding is easily broken at higher speeds, causing bigger materials to be removed from the work piece. The high impact energy is directly transferred to the composite, resulting in a faster erosion rate. The surface contact area of the eroded particles has a direct impact on the erosion at lower angles. At higher velocity, the impingement of erodent particles is also witnessed on the composite surface. The greater the angle, the greater the resistance to the erosion. This is because the erodent particles on the composite surface provide less impact stress. The microscopic and 3D profilometer images for varying tow sizes of 5k, 10k, and 15k are depicted in Figure 8, Figure 9, and Figure 10, respectively.

The eroded particles supplied at lower speeds did not penetrate the composite surface. The lower the collision force, the harder the traces on the surface, yet there was no sign of fibre breaking or pull-out. The microscopic view shows the fibre’s strong interfacial connection with the matrix. The passage of erodent particles on the composite caused surface waviness (flow trace), which prevented the surface from degrading.

## 4. Conclusions

A carbon fibre-reinforced polymer composite with varying tow sizes has been fabricated and the erosion behaviour has been determined. The observations are summarised as follows:

- The strength and the hardness of the composite increased to the maximum value for the increment of the tow scale from 5k to 10k, and then it tended to decrease.
- When the effect of erodent velocity was observed, it was higher, and the erosion rate and the surface roughness of the composite were also higher.
- The minimum erosion rate and a good surface finish were noticed at a higher impingement angle of 90 degrees, whereas the minimum impact angle of 30 degrees led to higher erosion rates and a poor surface finish.
- The erosion rate and the surface roughness of the composite were maximum for the 10k tow size fibre-reinforced polymer composite, whereas the 15k tow size fibre-reinforced polymer composite possessed an improved surface finish with a comparatively lower erosion rate.
- The eroded particles supplied at lower speeds did not penetrate the composite surface. The lower the collision force, the harder the traces on the surface, yet there was no sign of fibre breaking or pull-out.
- The passage of erodent particles on the composite caused surface waviness (flow trace), which prevented the surface from degrading.
- In the future, the same study can be extended to determine the erosion performance for varying percentages of fibre reinforcements and other fibre-reinforced polymer composites.

## Figures and Tables

**Table 1:** Typical properties of carbon fibre and fabric parameters.

Filament Count	Yield (g/1000m)	Tensile Strength (MPa)	Tensile Modulus (GPa)	Density (g/cm^3^)	Strain (%)
3K	200	3800	240	1.78	1.6
6K	400	3800	240	1.78	1.6

**Table 2:** Control factors and their levels.

SNo.	Parameters	Unit	Level/Range
1	Impact angle	degree	30, 60, 90
2	Erodent velocity	m/s	72, 100, 129
3	Tow size	Counts	5K, 10K, and 15K
4	Standoff distance	mm	10
5	Discharge rate	g/min	3.3

**Table 3:** Properties of the materials.

SNo.	Specimen	Tensile Strength (N/mm^2^)	Flexural Strength (N/mm^2^)	Impact Strength (J/m)	Hardness (HRN)	Density (g/mm^3^)
1	5K	441.94	724.46	34	112	1.515
2	10K	450.11	794.23	36	116	1.593
3	15K	379.48	751.80	36	112	1.489

**Table 4:** Experimental results.

SNo.	Tow	Angle (Degree)	Velocity (m/s)	Erosion Rate × 10^−5^ (g/g)	Roughness, R_a_ (μm)
1	5K	30	72	8.1818	4.314
2	5K	60	100	16.9697	4.519
3	5K	90	129	8.4848	3.669
4	10K	30	72	11.2121	6.938
5	10K	60	100	27.2727	6.514
6	10K	90	129	12.4242	6.540
7	15K	30	72	25.1515	8.685
8	15K	60	100	11.2121	2.237
9	15K	90	129	24.2424	4.262

**Table 5:** Response table for erosion rate.

Level	Tow Size	Angle	Velocity
1	−20.47	−22.42	−20.38
2	−23.86	−24.77	−24.43
3	−25.57	−22.72	−25.10
Delta	5.09	2.35	4.72
Rank	1	3	2

**Table 6:** ANOVA results for erosion rate.

Factors	Adj SS	DOF	Adj MS	% of Contribution	F-Value
Tow	40.4262	2	20.2131	45.23343	29,111.66
Angle	9.815	2	4.9075	10.98214	7067.965
Velocity	39.1298	2	19.5649	43.78287	28,178.1
Error	0.001389	2	0.000694	0.001554	
Total	89.37239	8	44.68619		

**Table 7:** Response table for R_a._

Level	Tow Size	Angle	Velocity
1	−12.36	−21.22	−12.00
2	−16.47	−12.12	−14.17
3	−17.91	−13.40	−20.57
Delta	5.55	9.10	8.57
Rank	3	1	2

**Table 8:** ANOVA results for R_a_.

Factors	Adj SS	DOF	Adj MS	% of Contribution	F-Value
Tow	49.7682	2	24.8841	15.82534	35,839.01
Angle	145.6008	2	72.8004	46.29829	104,849.9
Velocity	119.1138	2	59.5569	37.87593	85,776.07
Error	0.001389	2	0.000694	0.000442	
Total	314.4842	8	157.2421		

## Data Availability

Not applicable.
